# ^89^Zr-labeled CEA-targeted IL-2 variant immunocytokine in patients with solid tumors: CEA-mediated tumor accumulation and role of IL-2 receptor-binding

**DOI:** 10.18632/oncotarget.25343

**Published:** 2018-05-15

**Authors:** Emilie M.J. van Brummelen, Marc C. Huisman, Linda J. de Wit-van der Veen, Tapan K. Nayak, Marcel P.M. Stokkel, Emma R. Mulder, Otto S. Hoekstra, Danielle J. Vugts, Guus A.M.S. Van Dongen, Henk M. Verheul, Stefan Evers, Jean J. L. Tessier, Jose Saro, Jan H.M. Schellens, C. Willemien Menke-van der Houven van Oordt

**Affiliations:** ^1^ The Netherlands Cancer Institute, Amsterdam, The Netherlands; ^2^ VU University Medical Center/Cancer Centre, Amsterdam, The Netherlands; ^3^ Roche Pharma Research and Early Development, Roche Innovation Center, Basel, Switzerland; ^4^ Roche Pharma Research and Early Development, Roche Innovation Center, Zurich, Switzerland; ^5^ Utrecht Institute of Pharmaceutical Sciences (UIPS), Utrecht, The Netherlands; ^6^ Centre for Human Drug Research, Leiden, The Netherlands

**Keywords:** immunocytokine, interleukin-2, ^89^Zr, biodistribution, immuno-PET

## Abstract

Cergutuzumab amunaleukin (CEA-IL2v) is an immunocytokine directed against carcinoembryonic antigen (CEA) containing an IL2v-moiety with abolished IL-2 receptor (IL-2R) α binding. We describe the biodistribution and tumor accumulation of ^89^Zr-labeled CEA-IL2v. Twenty-four patients with advanced solid CEA positive (CEA+) or negative (CEA−) tumors received CEA-IL2v 6 mg (4 CEA+; 3 CEA−), 20 mg (9 CEA+), or 30 mg (4 CEA+; 4 CEA−) biweekly. In cycle 1, 2 mg of the total dose comprised ^89^Zr-CEA-IL2v (50 MBq) and serial ^89^Zr-PET imaging was conducted. Four CEA+ patients with visually confirmed ^89^Zr-CEA-IL2v tumor accumulation at 20 mg had repeated ^89^Zr-PET imaging during cycle 4. ^89^Zr-CEA-IL2v immuno-PET demonstrated preferential drug accumulation in CEA+ tumors (%ID/mL_peak_ CEA− 3.6 × 10^−3^ vs. CEA+ 6.7 ×∙10^−3^). There was a non-significant trend towards dose-dependent tumor uptake, with higher uptake at doses ≥20 mg. Biodistribution was dose- and CEA-independent with major accumulation in lymphoid tissue compatible with IL-2R binding. Reduced exposure and reduced tumor accumulation (%ID/mL_peak_ 57% lower) on cycle 4 vs. cycle 1 was consistent with peripheral expansion of immune cells. The findings of this immune PET imaging study with ^89^Zr-CEA-IL2v support the therapeutic concept of CEA-IL2v, confirming selective and targeted tumor accumulation with this novel immunocytokine.

## INTRODUCTION

Local immune suppression has been recognized as an emerging hallmark of many types of cancer. Stimulation of the immune system has proven to induce significant anti-tumor immune responses in several types of cancer including non-small cell lung, head and neck, renal cell, colorectal cancer and melanoma [1, 2]. Since the early 90s immuno-stimulation with interleukin-2 (IL-2) has been used to treat melanoma and renal cell carcinomas [3]. However, therapeutic use of IL-2 has been limited by its short half-life and severe toxicities such as vascular-leak syndrome, acute respiratory disorders, and hypotension [4]. Additionally, IL-2 activates regulatory T cells (T_regs_), which has an immunosuppressive effect. Both activation of T_regs_ and (pulmonary) vascular leakage are considered to be mediated by binding of IL-2 to IL-2-receptor alpha (IL-2Rα/CD25) [5]. This high-affinity receptor subunit is preferentially expressed on T_regs_ and endothelial cells, but it is not required for effector T-cell activation and expansion, which occurs primarily through the IL-2Rβ and IL-2Rγ subunits [6].

The targeted immunocytokine cergutuzumab amunaleukin (CEA-IL2v) has been designed to overcome the limitations of IL-2 therapy and is currently being developed in combination with the anti-PD-L1 antibody atezolizumab in CEA-expressing solid tumors (NCT02350673). CEA-IL2v consists of a carcinoembryonic antigen (CEA)-targeted IgG_1_-antibody fused to an engineered IL-2 variant (IL2v) with abolished IL-2Rα binding. CEA-IL2v is designed to induce a local immune response in the tumor by binding preferentially to CEA-expressing tumor cells while avoiding activation of T_regs_ due to reduced IL-2Rα binding, which was confirmed in preclinical experiments [7]. CEA is an attractive target for anticancer therapy because of its widespread expression on tumors. CEA is normally expressed during embryonal development and on healthy colon mucosa and is present as soluble CEA in the circulation. Nearly all colorectal, gastric, and pancreatic cancers overexpress CEA as well as 70% of the non-small cell lung cancers and 50% of breast cancers [8, 9]. To avoid trapping to soluble CEA, CEA-IL2v contains a membrane-proximal CEA target epitope which does not recognize soluble CEA [7]. Taken together, these characteristics support the potential of CEA-IL2v to preferentially activate immune effector cells over T_regs_ cells with a more favorable pharmacokinetic (PK), biodistribution, and tolerability profile compared with existing IL-2 therapy options.

Measuring the *in vivo* biodistribution of a monoclonal antibody (mAb) can provide valuable evidence to confirm the hypothesized mode of action of a novel targeted therapy and to support drug development decisions. Zirconium-89 (^89^Zr) labeling of a mAb and subsequent positron emission tomography (PET) is a non-invasive tool for *in vivo* visualization and quantification of mAbs [10]. Both target expression and tumor targeting of a novel mAb can be visualized using ^89^Zr-immuno-PET. Quantitative assessment of serial PET images enables drug PK and accumulation within tumor tissue to be calculated. Here we report the results of a ^89^Zr-immuno-PET substudy, as part of the first-in-human phase I dose-escalation study of CEA-IL2v (NCT02004106), that investigated the biodistribution and tumor accumulation of ^89^Zr-CEA-IL2v in patients with CEA positive (CEA+) and CEA negative (CEA−) tumors. Our data show that accumulation of CEA-IL2v in tumor tissue is mediated by CEA binding with a non-significant trend towards dose-dependent uptake, compared with uptake in non-tumor lymphoid tissue which is independent of dose. We also show how ^89^Zr-immuno-PET enabled assessment of CEA-IL2v exposure *in vivo* within tumor tissue. These data have since been used in the development of a PK/pharmacodynamic (PD) mathematical model that will inform the selection of optimal dosing and drug scheduling in later stage clinical trials [11]. We also discuss the added value of repeat imaging after multiple doses of CEA-IL2v, which revealed changes in biodistribution.

## RESULTS

### Patients and treatment

**Figure 1:**
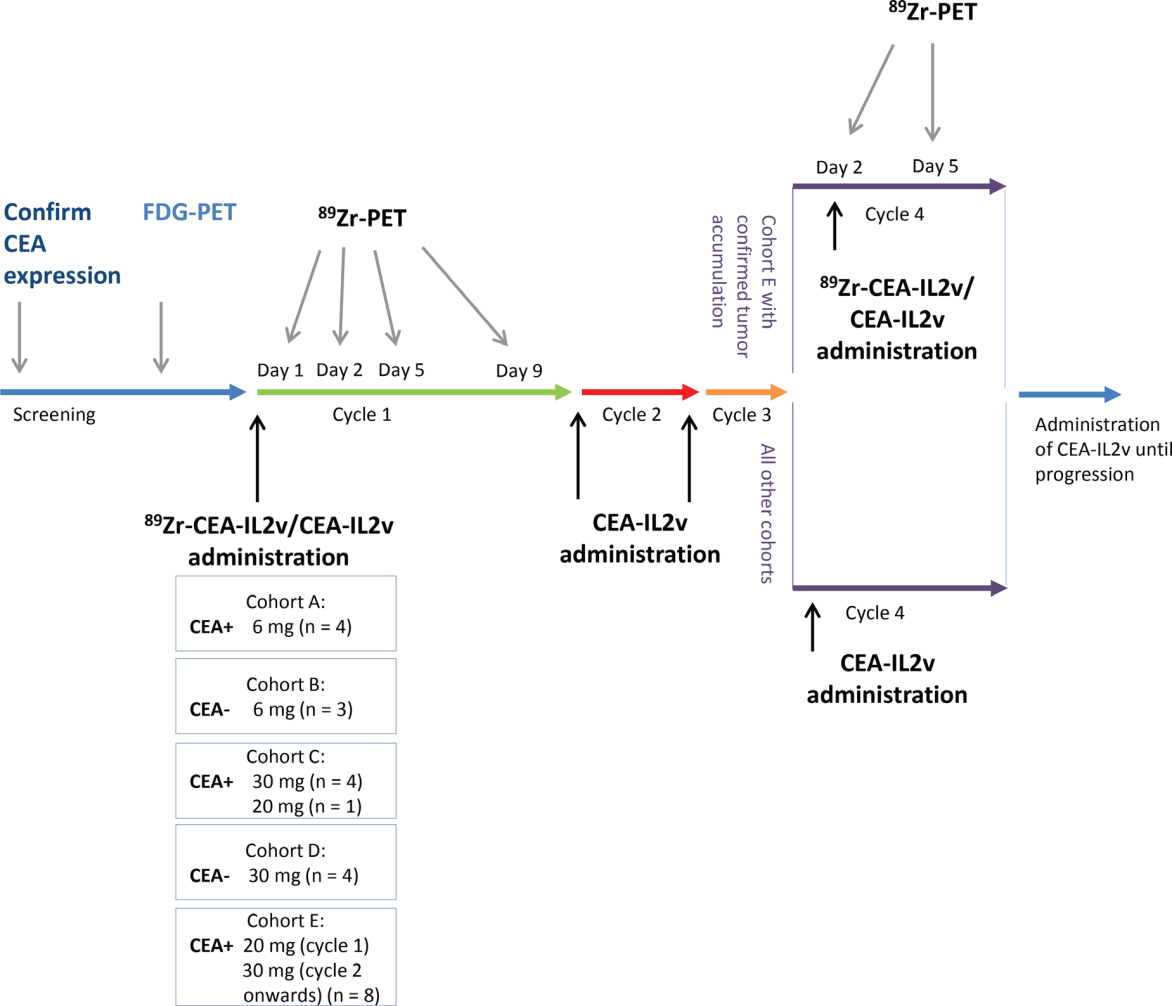
Study design and assessments Patients were assigned to one of five cohorts: cohorts A (CEA+) and B (CEA−) received CEA-IL2v 6 mg, cohorts C (CEA+) and D (CEA−) received 30 mg CEA-IL2v, and cohort E (CEA+) received 20 mg in cycle 1 and 30 mg in cycle 2 and thereafter. Unlabeled CEA-IL2v was administered intravenously over 2 hours on day 1 with 2 mg of the total dose subsequently administered as radiolabeled ^89^Zr-CEA-IL2v [50 MBq]. ^89^Zr-PET scans were conducted for each patient during cycle 1 on day 1 (2 hours p.i.), day 2 (24 hours p.i.), day 5 (96 hours p.i.), and day 9 (192 hours p.i.) and on day 1 of cycle 4 with subsequent ^89^Zr-PET scans at day 2 (24 hours p.i.) and 5 (96 hours p.i.).

Between June 2014 and March 2016, 24 patients were enrolled. Primary CEA+ (*n =* 17) tumors comprised colorectal cancer (*n =* 11), non-small cell lung cancer (*n =* 4), salivary gland cancer (*n =* 1), and gastric cancer (*n =* 1). CEA− tumors (*n =* 7) were renal cell cancer (*n =* 3), melanoma (*n =* 2), pancreatic cancer (*n =* 1), and ovarian cancer (*n =* 1). Baseline patient characteristics are summarized in Supplementary Table 1. All patients underwent ^89^Zr-CEA-IL2v administration and subsequent PET imaging. Patients were treated in all cycles with 6 mg (cohort A; CEA+ *n =* 4, cohort B; CEA− *n =* 3); 30 mg (cohort C; CEA+ *n =* 4, cohort D; CEA− *n =* 4), or 20 mg (cohort C; CEA+ *n =* 1); or 20 mg in cycle 1 and 30 mg in cycle 2 and onwards (cohort E; CEA+ *n =* 8; Figure 1). One patient in cohort E was not evaluable for assessment of tumor accumulation due to lack of extrahepatic ^18^F-FDG-PET positive tumor lesions; however, this patient was evaluable for biodistribution analyses. Six of the remaining patients in cohort E showed tumor accumulation after cycle 1, and four of these had a second ^89^Zr-CEA-IL2v administration and ^89^Zr-PET imaging on treatment in cycle 4. The two other patients could not continue study procedures due to previously unknown brain metastases and progressive disease, respectively.

### Tumor accumulation

**Figure 2:**
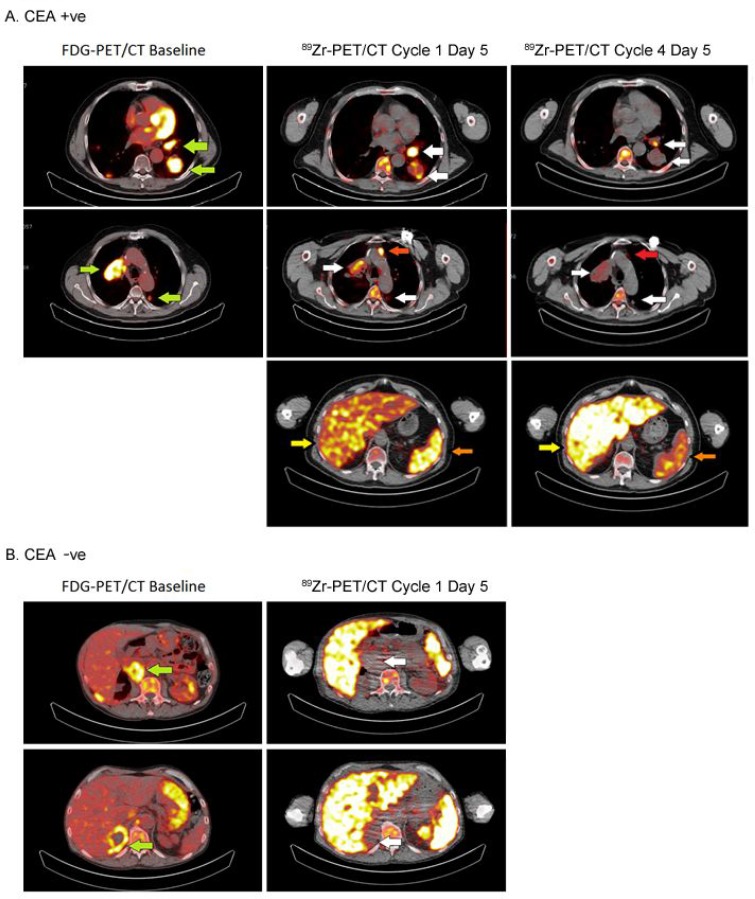
Tumor accumulation in a CEA+ and a CEA− patient treated with 20 mg and 30 mg of CEA-IL2v, respectively Images of ^89^Zr-PET at cycle 1, day 5 (96 hours) are shown (left) and tumor lesions were identified by ^18^F-FDG-PET at screening (right, green arrows). In the patient with CEA+ colorectal cancer (**A**), ^89^Zr-CEA-IL2v accumulation was observed in the left and right hilar lymph nodes, the left dorsal lung lesion (accumulation indicated by white arrows) and a non-pathological lymph node (accumulation indicated by red arrow) in the CEA+ patient. For the CEA− patient (**B**), the adrenal gland and abdominal lymph node lesions were negative on ^89^Zr-PET. Part A also shows that accumulation of ^89^Zr-CEA-IL2v in tumor decreased between day 5 (96 hours) of cycle 1 and cycle 4. Following the first dose of 20 mg, this patient received 30 mg every 2 weeks for subsequent cycles as part of cohort E. Also spleen accumulation was decreased (orange arrow), while liver accumulation was increased (yellow arrow).

Of 24 patients included, 23 were evaluable for ^89^Zr-PET analysis of tumor accumulation on cycle 1, day 5 (96 hours post-injection [p.i.]). One patient from cohort E was excluded because the extrahepatic tumor lesion was ^18^F-FDG-negative and did not meet the criteria for evaluability. Evidence for CEA targeting of CEA-IL2v was apparent from visual analysis of PET images (Figure 2): tracer uptake was seen in tumor lesions in 14 out of 16 (88%) patients with CEA+ tumors and in four out of seven (57%) patients with CEA− tumors (Supplementary Table 2). There was a non-significant trend towards dose-dependent tumor uptake of ^89^Zr-CEA-IL2v in CEA+ patients. Tumor accumulation was observed in three out of four (75%) CEA+ patients treated at 6 mg, seven out of eight (88%) evaluable patients at 20 mg, and all four (100%) patients at 30 mg. At the 6 mg dose, tumor uptake was below the limit of quantification for CEA− tumors (data not shown). For patients treated at 30 mg (Supplementary Figure 1), we observed a non-significant difference in uptake between CEA+ and CEA− patients. While some tumor uptake was observed in CEA− patients (cohort D), the extent was lower than for CEA+ patients (cohort C mean %ID/mL_peak_ CEA− 3.6 ×∙10^–3^ ± 1.7 ×∙10^–3^ vs. CEA+ 6.7 ×∙10^–3^ ± 5.9 ×∙10^–3^, *P* = 0.15). Tracer uptake appeared to increase over time in CEA+ tumors, indicating accumulation of antibody within tumor tissue, whereas uptake in CEA− tumors remained relatively constant. Importantly, a clear difference was apparent between levels of ^89^Zr-CEA-IL2v measured in the tumor tissue compared with the blood (Supplementary Figure 1).

**Figure 3:**
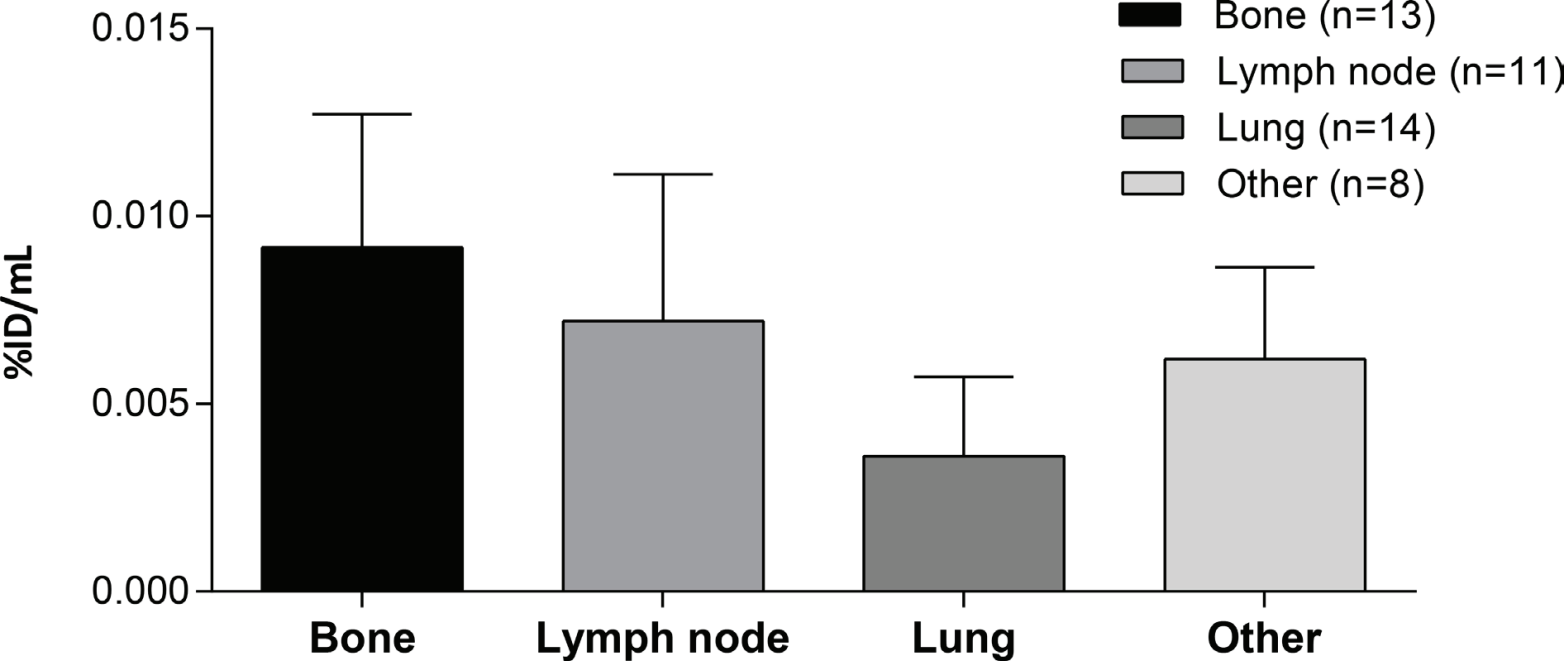
Tumor accumulation according to lesion location Tumor targeting at cycle 1, day 5 (96 hours) of different lesions from CEA+ patients treated with doses ≥20 mg in peak percentage of injected dose per mL (%ID/mL_peak_) with the corresponding number of lesions per group (n). ‘Other’ includes parotid, coecum, cerebellum, pelvic, adrenal, and soft tissue lesions.

Tumor accumulation differed depending on tumor lesion location (Figure 3): the highest accumulation was found in bone metastases and lymph nodes with lower accumulation in pulmonary lesions. Tumor lesions located in the colorectal tract and liver could not be accurately quantified due to high background accumulation (hepatobiliary excretion resulting in high local concentrations in liver and fecal matter) with resulting spill-in effects. The majority of visually positive tumor lesions were positive at all timepoints, with only five tumor lesions (three lymph nodes, one lung, and one liver) visually positive on one timepoint.

### Biodistribution in non-tumor tissue

**Figure 4:**
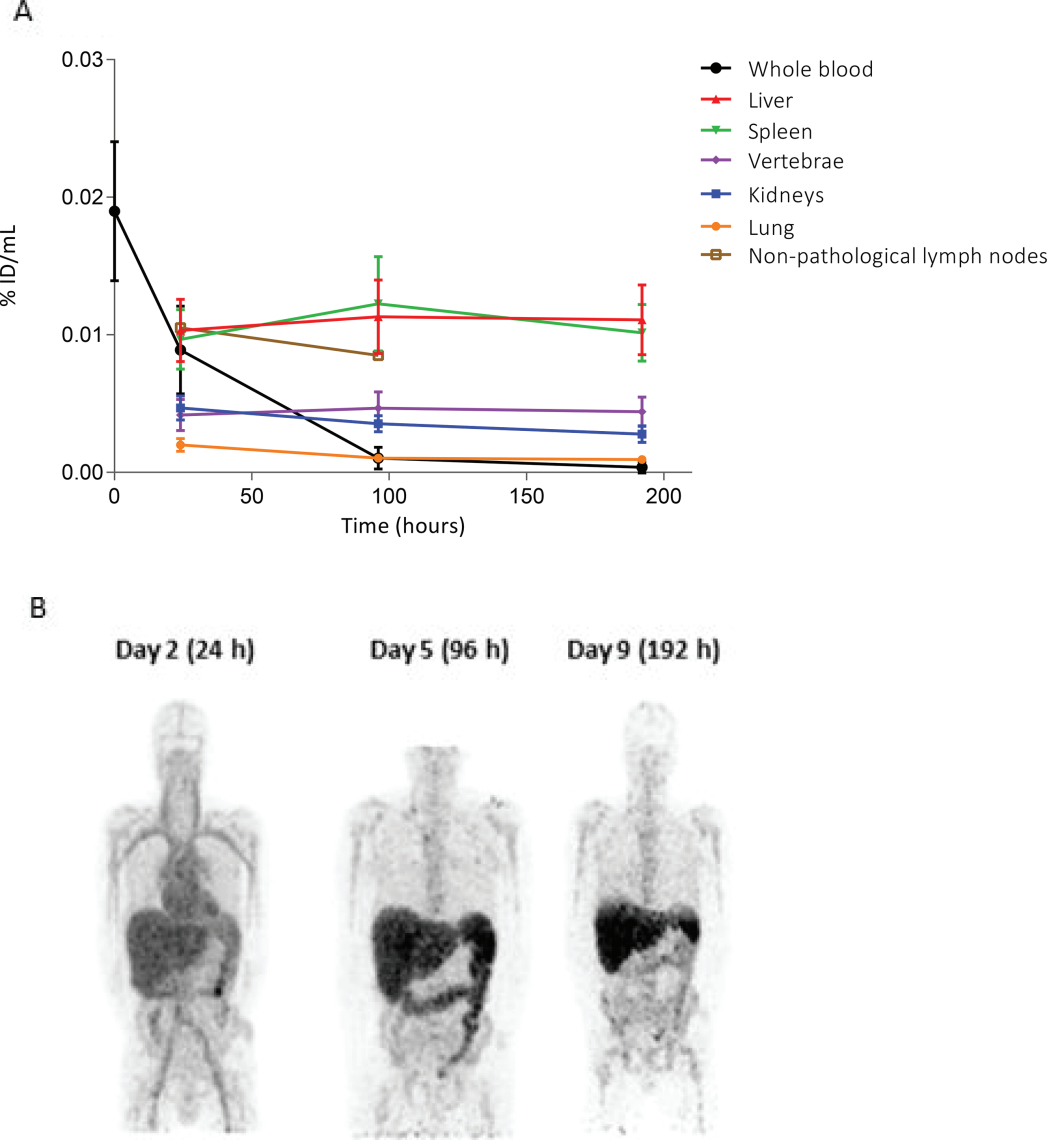
Biodistribution of ^89^Zr-CEA-IL2v (**A**) Distribution of ^89^Zr-CEA-IL2v in non-tumor tissues as percentage of injected dose per milliliter (%ID/mL) over time in cycle 1 assessed on day 2 (24 hours), day 5 (96 hours), and day 9 (192 hours). Data for all patients are combined (*n* = 24). Organ accumulation is shown as %ID/mL mean, whole blood as %ID/mL, and non-pathological lymph nodes as %ID/mL peak. (**B**) Biodistribution on day 2 (24 hours), day 5 (96 hours), and day 9 (192 hours) post-infusion in a representative maximum intensity projection of a CEA− renal cell carcinoma patient treated with 30 mg ^89^Zr-CEA-IL2v. At day 2, homogeneous tracer distribution in the circulation is observed. At day 5, increased hepatic accumulation and hepatobiliary excretion is observed, which is decreased again at day 9.

^89^Zr-CEA-IL2v blood levels and biodistribution in non-tumor tissues in cycle 1 are presented in Figure 4, showing homogeneous tracer distribution in the circulation at day 2, increasing hepatic and splenic uptake at day 5 and decreasing uptake at day 9, partially due to subsequent excretion. Immediately after infusion (*t* = 0), the mean recovered dose of CEA-IL2v was 19%/L (range: 8–31%/L) in whole blood and 33%/L (range: 17–53%/L) in serum. ^89^Zr-CEA-IL2v was cleared from the blood with an apparent elimination half-life of 34 hours (range: 9–53 hours) independent of dose. Pharmacokinetic curves of ^89^Zr-CEA-IL2v showed similar patterns to those of unlabeled CEA-IL2v although the peak concentrations on the first day of infusion were less pronounced for unlabeled CEA-IL2v (Supplementary Figure 2).

Across all treatment groups, tissue accumulation in non-malignant tissue on day 2 was highest in the liver and spleen, where it remained high at day 5 and day 9 (Figure 4A). High ^89^Zr accumulation was also observed in non-pathological (^18^F-FDG negative) lymph nodes in eight patients from different cohorts. Seventeen ^89^Zr-positive non-pathological lymph nodes were identified in total. As expected, non-pathological lymph nodes were not evaluable for quantification due to size <15 mm, consistent with RECIST 1.1 criteria. Lower uptake on day 2 was observed in the kidney, vertebrae, and lung and tracer accumulation in these organs decreased slightly from day 5 to day 9. Biodistribution was generally independent of dose and CEA status (Supplementary Table 3): high uptake of ^89^Zr-CEA-IL2v was seen in the spleen, liver, and non-pathological lymph nodes at all doses and for both CEA+ and CEA− patients. The only exception was splenic uptake, which was significantly lower in the patients treated with 30 mg compared to the other dose-cohorts for both CEA+ and CEA− patients (Supplementary Table 3), although the clinical relevance of this significant discrepancy is unclear. Again, a marked difference was observed between ^89^Zr-CEA-IL2v PK measured in the blood compared with the different tissues (Figure 4A).

Elimination occurred via hepatobiliary excretion, as can be seen on the day 5 ^89^Zr-PET scan (Figure 4B).

### Changes in biodistribution and tumor accumulation during treatment

**Figure 5:**
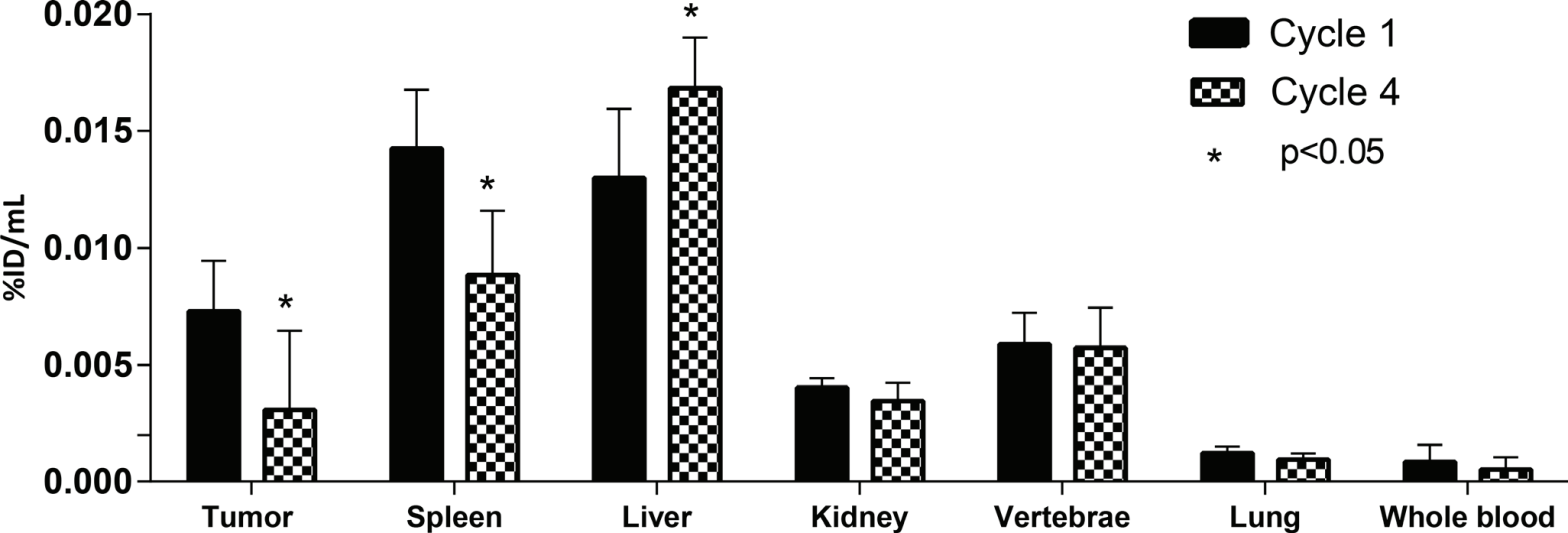
Tumor, organ and whole blood ^89^Zr-CEA-IL2v accumulation during cycle 1 and cycle 4 of treatment Accumulation of ^89^Zr-CEA-IL2v in tumors, spleen, kidney, vertebrae, lung, and whole blood in cycle 1, day 5 (96 hours) post-infusion versus cycle 4, day 5 (96 hours) post-infusion as percentage of injected dose per mL (%ID/mL) (whole blood) or as %ID/mL mean (organs) or %ID/mL peak (tumor). The asterisk (^*^) indicates a statistically significant difference (*p* < 0.05), assessed by a two-tailed paired Students’ *T*-test. Data are for patients with CEA+ tumors from cohort E (*n* = 4) treated with 20 mg for the first cycle and 30 mg thereafter.

We also investigated the effect of multiple administrations of CEA-IL2v on drug biodistribution and accumulation. Four patients in cohort E had two administrations of ^89^Zr-CEA-IL2v, one at treatment start in cycle 1 (total CEA-IL2v dose 20 mg) and one on-treatment in cycle 4 (after three further doses of 30 mg CEA-IL2v [total dose]). Exposure to ^89^Zr-CEA-IL2v on cycle 4, day 5 was 23% lower than on cycle 1, day 5, as evidenced by a decreased area under the serum concentration-time curve (0–192 hours) (Supplementary Figures 2 and 3). Tumor accumulation, as defined with %ID/mL_peak_, was 57% lower in cycle 4 than in cycle 1 (Figure 2A and Figure 5). Fifteen lesions in the four patients from cohort E had visually confirmed accumulation in cycle 1, whereas only nine lesions showed accumulation in cycle 4. Tissue distribution (%ID/mL_mean_) in non-malignant organs also changed on-treatment, with 38% lower accumulation in the spleen (*P* = 0.001) and 29% higher hepatic accumulation (*P* = 0.02) in cycle 4 compared with cycle 1 (Figure 2). ^89^Zr-CEA-IL2v accumulation in the vertebrae, kidney, and lung did not change with treatment.

### Safety and efficacy

The most frequently observed adverse events related to treatment with CEA-IL2v in this substudy were infusion related reactions (63%), pyrexia (54%), fatigue and nausea (46% for both). Four patients discontinued treatment due to adverse events (pain, dyspnea, pulmonary hypertension and diarrhea). No adverse events specifically due to radiolabeled CEA-IL2v were observed. Anti-CEA-IL2v anti-drug antibodies (ADAs) were detected in 14 out of 20 (70%) patients with evaluable samples; in most cases these patients were ADA positive at the first on-treatment assessment (42 days).

Out of 21 patients evaluable for response, the best response was stable disease in three patients (14%) and progressive disease in 18 patients (86%). In this small study, no relation between drug tumor accumulation and response was identified.

## DISCUSSION

In this exploratory clinical immuno-PET study, we showed that CEA-IL2v targets CEA+ tumors preferentially with a non-significant trend towards dose-dependent CEA-mediated tumor accumulation. After multiple drug administrations, we observed changes in ^89^Zr-CEA-IL2v whole body distribution and tumor uptake, although the number of evaluable patients was small. In addition, ^89^Zr-immuno PET revealed differences in drug PK in tumor tissue compared with blood PK, which will be relevant to the selection of appropriate drug schedules for subsequent clinical trials.

At baseline, tumors were classified as CEA+ and CEA− based on archival material from the primary tumor or a metastasis. Tumor drug accumulation was higher and more consistent in CEA+ patients compared with CEA− patients (Figure 2; Supplementary Figure 1), consistent with the mode of action of CEA-IL2v (i.e., tumor targeting mediated by CEA binding). Of note, statistical analyses in this study were performed using only quantifiable lesions (the most widely-used practice in nuclear imaging), which may have reduced the difference between CEA+ and CEA− patients (the latter group having fewer quantifiable lesions). CEA-mediated tumor accumulation has been reported before in several clinical trials with anti-CEA antibodies [12–18], mainly in colorectal, liver, bone, thyroid, and lymph node lesions; however, none of the trials confirmed tumor CEA expression with immunohistochemistry. For other bispecific CEA constructs consisting of anti-CEA-CD3 and anti-CEA-B7, CEA-mediated accumulation was confirmed only in preclinical experiments [19–21]. In our study, tumor accumulation was still present at day 9 while most of the drug was cleared from blood, confirming retention of the drug in the tumor. This retention was most pronounced in the cohort treated with the highest dose (30 mg).

Limited drug uptake was observed at the highest dose (30 mg CEA-IL2v) in CEA− tumors, albeit much less than for CEA+ tumors. Although intratumoral heterogeneity of CEA expression is not well described in literature [22], published data [23] suggests that it is highly unlikely that tumors categorized as CEA− tumors by immunohistochemistry may have expressed some CEA. Therefore, the tumor accumulation observed in CEA− tumors is probably due to other mechanisms. We hypothesize that the drug uptake in CEA− tumors is primarily caused by CEA-IL2v binding to IL-2 receptor present on tumor infiltrating lymphocytes similarly to what has been reported for IL-2 therapy in melanoma, head and neck- and renal cell carcinoma [24–26]. This hypothesis is supported by the observation that, independently of tumor CEA status, ^89^Zr-CEA-IL2v accumulation in all cohorts was high in non-tumor lymphoid organs such as spleen and some non-pathological lymph nodes which also contain IL-2R-expressing immune cells (NK cells, and CD4^+^/CD8^+^ T cells). This mechanism could also explain the potentially dose-dependent decrease seen in splenic uptake (Supplementary Table 3). At the 6 mg dose, the spleen is believed to be acting as an IL-2R sink organ for CEA-IL2v and sequestering most of the dose (including most of the ^89^Zr-labeled fraction). This is consistent with the lack of difference in tumor uptake between CEA+ and CEA− tumors at the 6 mg dose. Higher doses of drug may saturate this sink organ leaving sufficient amounts of ^89^Zr-labeled CEA-IL2v to target CEA within tumor tissue, thereby simultaneously reducing splenic uptake. Although lymph node accumulation is generally minor for antibodies [27], it was pronounced for ^89^Zr- CEA-IL2v both in pathological and non-pathological lymph nodes [24–26]. The high accumulation seen in pathological lymph nodes may reflect a combination of CEA-targeting and IL-2R binding on immune cells. Due to the small size of non-pathological lymph nodes, it was nevertheless not possible to reliably quantify non-pathological nodes for comparison.

Like other antibodies, ^89^Zr-CEA-IL2v distributed to highly perfused tissues such as liver, spleen and bone marrow and to a lesser extent to lung, kidneys and lymph nodes [27]. In lung, kidney and bone marrow, CEA-IL2v accumulation seemed directly related to the blood concentration. Lower accumulation in pulmonary lesions may be a consequence in part of pulmonary motion, which has been shown to cause deterioration in PET image quality and lower standardized uptake value (SUV) estimates in lung lesions with other tracers, such as ^18^F-FDG [28]. Accumulation in the liver was considered to be related to antibody metabolism. The relatively high uptake of ^89^Zr-CEA-IL2v in bone lesions compared to other tumor locations may be related to the bone-seeking characteristic of ^89^Zr [29]. However, based on the stability of the conjugation method [30] and product (Roche, unpublished internal data) we do not expect that significant levels of free ^89^Zr are present in circulation, which is supported by the fact that no significant accumulation in healthy bone was observed. As an alternative explanation, bone metastases may be more easily accessible for antibodies, which has also been observed for other ^89^Zr-labeled antibodies [31].

Importantly, ^89^Zr-immuno-PET enabled us to quantitatively measure drug exposure *in vivo* within tumor tissue. This analysis revealed differences between tumor PK and blood PK and showed that CEA-IL2v levels in the blood do not accurately reflect the level of drug within the tumor. These data can enable drug schedules to be optimized based on exposure and accumulation within the tumor, as opposed to using conventional blood PK data obtained from measurement of non-labeled antibody to guide schedule selection. Indeed, a comprehensive PK/PD mathematical model has been created that incorporates the expansion of IL-2R positive target cells at multiple doses levels, different schedules of CEA-IL2v, and tumor uptake using imaging data from this study [11]. The model allows the prediction of patient-specific drug uptake into tumors and will be valuable in proposing CEA-IL2v dosing and scheduling to be tested in early dose-finding clinical studies.

The overall blood PK profile of ^89^Zr–CEA-IL2v was comparable to unlabeled CEA-IL2v. However, on day 1 shortly after infusion we observed relatively low levels of unlabeled CEA-IL2v whereas a pronounced ^89^Zr-CEA-IL2v peak concentration was present (Supplementary Figure 2). This may be due to a difference in sensitivity of the analytical methods; detection of ^89^Zr-CEA-IL2v was based on radioactivity measurements whereas unlabeled CEA-IL2v was detected by ELISA. Also, the interval between administration of unlabeled and labeled ^89^Zr-CEA-IL2v could result in discrepancies in PK profiles. Based on preclinical PK data for CEA-IL2v [32] and previous studies with anti-CEA antibodies [13], an increased clearance at higher doses and in CEA+ cohorts could have been expected. This was not observed in our study, possibly due to small cohort sizes and high intra-patient variability in PK data [18, 33].

Uniquely, this study incorporated on-treatment ^89^Zr-PET imaging in cycle 4 which enabled us to identify changes in tissue biodistribution over time, although the number of evaluable patients was small (*n =* 4). Reduced exposure to ^89^Zr-CEA-IL2v was observed following multiple CEA-IL2v treatments and reduced accumulation in the tumor and spleen at cycle 4 compared with cycle 1 was also observed, while uptake in the liver was increased. The reduction in exposure at later cycles is consistent with a treatment-induced expansion of IL-2R expressing circulating immune cells leading to increased binding of CEA-IL2v to immune cells, thus increasing clearance from the circulation [34]. This would reduce the level of CEA-IL2v available for tumor uptake. The reduction in spleen accumulation in cycle 4 compared with cycle 1 (Figure 2A and Figure 5), despite lymphocyte expansion, may also reflect IL-2R occupancy with unlabeled CEA-IL2v leading to reduced IL-2R-bound ^89^Zr-CEA-IL2v. Similarly, occupation of CEA binding sites within the tumor with unlabeled CEA-IL2v may explain the decreased tumor accumulation observed at cycle 4 compared with cycle 1 (Figure 2A and Figure 5); however, the short terminal half-life and relatively low dose of CEA-IL2v would argue against this [16]. The simultaneously increased liver accumulation in cycle 4 may be explained by increased ^89^Zr-CEA-IL2v metabolism in the liver. Liver metabolism could be enhanced by ADAs [35, 36] which were found in 70% of the patients in this study. In addition, these ADAs could hinder binding to tumor cells or lymphocytes resulting in decreased spleen and tumor accumulation by shielding the IL-2v or the anti-CEA antibody moiety. However, among the four patients who had on-treatment ^89^Zr-PET scans in cycle 4, only one had detectable ADAs at the time of imaging, which leaves the mechanism of the observed alterations in biodistribution in the other three, ADA-negative patients unexplained.

## MATERIALS AND METHODS

### Patient population

Patients with advanced and/or metastatic solid tumors without standard treatment options were included if they had CEA+ tumors, defined as ≥20% of tumor cells with moderately intense staining by immunohistochemistry on archival or freshly obtained tumor material. Patients with CEA− tumors (defined as 0% staining) were also included as a control group to investigate IL2v mediated biodistribution. Staining for CEA was performed locally with a CEA31 mouse monoclonal IgG_1_ anti CD66/CEACAM5 antibody (Cell Marque #760-4594, Ventana Medical Systems, USA) using an in-house validated procedure. Other eligibility requirements were age ≥18 years, ≥ one tumor lesion accessible for a biopsy, ≥ one non-liver lesion assessable by ^89^Zr-PET imaging and radiologically measurable disease per Response Evaluation Criteria in Solid Tumors (RECIST) version 1.1, life expectancy of more than 12 weeks, World Health Organization (WHO)/Eastern Cooperative Oncology Group (ECOG) performance status of 0 or 1, and adequate organ function including hematological, renal, hepatic, and coagulation parameters. Exclusion criteria included history of current central nervous system tumors, active second malignancies, except non-melanoma skin cancer or cervical carcinoma *in situ*, active infections, uncontrolled concomitant diseases or mental illnesses which could affect protocol compliance, uncontrolled hypertension, pregnancy, HIV, major surgery and/or immunotherapies or immunosuppressive drugs within 28 days prior to start, hypersensitivity to the investigational drug, premedications (corticosteroids, antihistamines, paracetamol or 5-HT_3_ antagonists) or 2-(^18^F)-fluoro-2-deoxyglucose, concurrent therapy with investigational drugs, chronic or high-dose use of corticosteroids (>20 mg dexamethasone-equivalents), immunosuppressive therapy, baseline QTc interval >470 ms, bradycardia (<45 bpm) or tachycardia (>100 bpm), and wide-field radiotherapy within four weeks prior to start.

### Study overview

This study was conducted at VU University Medical Center (VUmc) Cancer Center and the Netherlands Cancer Institute in Amsterdam, as a substudy of the first-in-human trial with CEA-IL2v (NCT02004106). The study protocol and all amendments were approved by the local ethics committees. All patients provided written informed consent before any study procedure began. The study was conducted in accordance with the International Conference on Harmonization Good Clinical Practice Guideline with the ethical principles of the current version of Declaration of Helsinki and local regulatory guidelines.

### Study objectives

The primary objective of this imaging substudy was to investigate the *in vivo* biodistribution of radioactively labeled ^89^Zr-CEA-IL2v in patients who received different doses of CEA-IL2v. This included assessment of the extent and kinetics of tumor accumulation of ^89^Zr-CEA-IL2v measured by PET, the distribution of ^89^Zr-CEA-IL2v to lymphoid tissues and other organs, and investigation of potential differences between CEA+ and CEA− tumors with regards to ^89^Zr-CEA-IL2v tumor accumulation and biodistribution.

### Study design and procedures

The study design is shown in Figure 1. Patients with CEA+ or CEA− tumors were assigned to one of five cohorts: cohorts A (CEA+) and B (CEA−) received a total dose of CEA-IL2v of 6 mg and cohorts C (CEA+) and D (CEA−) received 30 mg CEA-IL2v. Based on emerging safety data from the phase I trial, the last patient in cohort C received 20 mg CEA-IL2v and patients in a subsequent cohort E (CEA+) received 20 mg in cycle 1 and 30 mg in cycle 2 and onwards. For the first CEA-IL2v dose (day 1, cycle 1), 2 mg of the total dose was given as radiolabeled ^89^Zr-CEA-IL2v (50 MBq). The total dose of unlabeled CEA-IL2v was administered intravenously over 2 hours on day 1 with subsequent administration of 2 mg ^89^Zr-CEA-IL2v (<2 hours after end of administration of unlabeled CEA-IL2v). A maximum of three ^89^Zr-PET scans per patient were conducted on day 1 (2 hours p.i. for dosimetry purposes in the first three patients only), day 2 (24 hours p.i.), day 5 (96 hours p.i.), and day 9 (192 hours p.i.). Patients in cohort E who had confirmed ^89^Zr-CEA-IL2v tumor accumulation by visual analysis after PET imaging in cycle 1 received a second administration of 2 mg/50 MBq ^89^Zr-CEA-IL2v as part of the total dose on day 1 of Cycle 4 with subsequent ^89^Zr-PET scans at day 2 (24 hours p.i.) and 5 (96 hours p.i.).

After completing the imaging procedures in cycle 1 (or cycle 1 and 4 for cohort E), patients continued treatment with CEA-IL2v given on day 1 of a 14 day cycle in the highest dose cohort that was cleared for safety until confirmed disease progression, death, unacceptable toxicity, withdrawal of consent, or investigators’ decision to stop.

### PET imaging procedures

CEA-IL2v was labeled at VUmc with ^89^Zr (BV Cyclotron VU, Amsterdam, the Netherlands) according to Good Manufacturing Practice (GMP) standards, as previously described [37]. 2 mg/50 MBq ^89^Zr-CEA-IL2v was infused over 10 minutes within 2 hours of infusion of unlabeled CEA-IL2v as part of the total CEA-IL2v dose. Whole body PET-low dose computed tomography (ldCT) and ^89^Zr-PET scans were acquired on a Gemini TF-64 or Ingenuity TF PET/CT scanner (Philips Healthcare, Best, the Netherlands). Scanners were EARL accredited, cross-calibrated and images were reconstructed as previously described [38]. ^18^F-FDG-PET scans were performed at baseline according to European Association of Nuclear Medicine (EANM) 2.0 guidelines [39] to identify evaluable malignant lesions for ^89^Zr-PET imaging.

### ^89^Zr-CEA-IL2v PET data analysis

Visual assessment of PET images to determine biodistribution and tumor accumulation was performed by three physicians experienced in PET image analysis (OSH/LWV/MST): ^89^Zr-PET data was first assessed alone to identify positive lesions, followed by combined assessment with ^18^F-FDG-PET and clinical history to confirm ^89^Zr-positive lesions as tumor lesions or non-pathological lesions, and to identify ^89^Zr-negative lesions. Readings were done by two teams (NKI and VuMC) independently and in case of disagreement the reading was repeated to obtain consensus. Visual tumor accumulation of ^89^Zr-CEA-IL2v was defined as visually enhanced accumulation exceeding local background. At a patient level, ^89^Zr-PET scans were considered positive if at least one non-hepatic tumor lesion showed ^89^Zr accumulation on the cycle 1, day 5 scan and one additional scan supporting consistent tumor accumulation. Tumor accumulation was quantified on day 5 because image quality was optimal with the lowest background activity, which is comparable to previous clinical studies with ^89^Zr-labeled antibodies [10, 13]. According to RECIST 1.1 criteria, only tumor lesions ≥10 mm (≥15 mm for lymph nodes) were identified as measurable tumor lesions. Only these measurable lesions were quantified to limit partial volume effects to <50% of variability in the recovered activity [38]. Smaller lesions were considered not evaluable for quantification. ^89^Zr- CEA-IL2v positive non-pathological (^18^F-FDG negative) lymph nodes were analyzed visually as quantification of lesions <15 mm is not reliable due to partial volume effects [38].

In all ^89^Zr-PET scans, volumes of interest (VOIs) of whole organs (liver, spleen, kidney, lung, bone marrow), and tumor lesions were manually delineated to derive percentage of injected dose per volume of tissue of interest (%ID/mL) as %ID/mL_mean_ for tissue and %ID/mL_peak_ for tumor to account for segmentation errors and background accumulation using in-house developed software [38]. PET results in tumor tissue were expressed as %ID/ml instead of SUV as this allowed a better comparison with blood counts (which were also expressed as %ID/ml), and use of SUV does not result in different conclusions. For lung, VOIs were semi-automatically defined on the ldCT and projected on the PET images. VOIs of the liver, spleen and kidney were manually delineated on the PET images themselves using the ldCT as reference. Fixed sized VOIs with volumes of 8.6 mL were placed on lumbar vertebrae on ldCT. Serum and whole blood ^89^Zr-CEA-IL2v concentrations were assessed by radioactivity measurements in a cross-calibrated gamma counter (Wizard 3, PerkinElmer, USA in the Netherlands Cancer Institute and Wallac Wizard 1480, PerkinElmer, USA in VUmc).

### Other study assessments

Baseline characteristics were collected including age, gender, Eastern Cooperative Oncology Group (ECOG) performance status and tumor type.

^89^Zr-CEA-IL2v pharmacokinetic and CEA-IL2v pharmacodynamic analyses were conducted in whole blood and serum collected via an intravenous catheter. Samples were taken before dosing on cycle 1, day 1; at the end of infusion; at 2 hours and 4 hours post-infusion; and on day 2, 5, and 9 at the same time as the PET scan. For patients in cohort E who received a second ^89^Zr-CEA-IL2v administration in cycle 4, samples were collected at the same timepoints in cycle 4. Serum drug (unlabeled CEA-IL2v) and anti-CEA-IL2v antibody concentrations were determined at a central laboratory by a validated ELISA method. The assay to detect serum drug concentration used a biotinylated mouse mAb directed against the idiotype of CEA-IL2v as capture reagent and digoxigenylated recombinant human IL-2R along with horseradish peroxidase conjugated anti-digoxigenin fragments as detection molecules. This bi-functional, target-binding competent assay has a sensitivity of 0.7 ng/mL in human serum. The bioanalytical method for the detection of anti-CEA-IL2v antibodies has been described previously [40]. Briefly, a bridging ELISA was used with biotinylated and digoxigenylated CEA-IL2v as capture/detection reagents in a three-tiered approach starting with ADA screening (tier 1) and following confirmation (tier 2) and titration assay (tier 3). Affinity-purified anti-idiotypic polyclonal antibodies directed against CEA-IL2v were used as a positive control. The sensitivity of the ELISA was 15.1 ng/mL for the used positive control.

Safety was assessed by Common Terminology Criteria for Adverse Events (CTCAE) v4.03, although the full safety profile was the objective of the separate phase I study and is therefore not extensively described in this paper. Response assessment was done according to RECIST 1.1 by CT at baseline and at 12 weeks, and every eight weeks thereafter. Laboratory checks and safety assessments were done throughout the study.

### Statistical analysis

Since the objectives of this study were purely descriptive, no formal statistical justification is provided for the sample size. Patients were evaluable for biodistribution imaging if they had at least one ^89^Zr-PET scan after administration of ^89^Zr-CEA-IL2v and for tumor accumulation if in addition at least one ^18^F-FDG positive (+) tumor lesion was ^89^Zr-PET assessable; patients with non-quantifiable lesions were not included. Patients were evaluable for safety and PK after administration of one dose of (unlabeled) CEA-IL2v. For efficacy analysis, one on-treatment tumor assessment was required. The relationship between organ accumulation, dose, and CEA status was analyzed by One Way Analysis of Variance (ANOVA). The difference between organ accumulation in cycle 1 and cycle 4 per patient was analyzed by a two-tailed paired Students’ *T*-test. All analyses were performed in R [41], software for statistical computing and graphics.

## CONCLUSIONS

In conclusion, this immuno-PET study supports the concept of this CEA tumor-targeting cytokine. ^89^Zr-immuno-PET provided unique information on drug uptake in tissues over time that could not be estimated from conventional assessment of blood PK. In particular, CEA-mediated tumor uptake was seen in CEA+ tumors at doses ≥20 mg but not at the lower dose investigated (6 mg). These data can be used to support optimal dose and schedule selection, based on CEA-IL2v exposure and accumulation within the tumor, rather than conventional blood PK data obtained from measurement of non-labeled antibody.

## SUPPLEMENTARY MATERIALS FIGURES AND TABLES



## Abbreviations

ADA: anti-drug antibody
CT: computed tomography
CTCAE: Common Terminology Criteria for Adverse Events
CEA: carcinoembryonic antigen
CEA-IL2v: cergutuzumab amunaleukin
ECOG: Eastern Cooperative Oncology Group
ELISA: enzyme-linked immunosorbent assay
FDG: fluorodeoxyglucose
IL-2: interleukin-2
IL-2Rα: IL-2-receptor alpha
%ID/mL: percentage of injected dose per volume of tissue of interest
ldCT: low dose computed tomography
mAb: monoclonal antibody
PD: pharmacodynamic
PET: positron emission tomography
p.i.: post-infusion
PK: pharmacokinetic
SUV: standardized uptake value
T_regs_: regulatory T cells
VOI: volumes of interest
VUmc: VU University Medical Center
WHO: World Health Organization
^89^Zr: Zirconium-89
