# Supplementary material for: 89Zr-labeled CEA-targeted IL-2 variant immunocytokine in patients with solid tumors: CEA-mediated tumor accumulation and role of IL-2 receptor-binding
Source: Oncotarget. 2018 May 15;9(37):24737–49. doi: 10.18632/oncotarget.25343 (PMC5973872; doi:10.18632/oncotarget.25343)
Supplement: Supplementary file 1 [file oncotarget-09-24737-s001.pdf]

^{89}Zr-labeled CEA− targeted IL-2 variant immunocytokine in patients with solid tumors: CEA− mediated tumor accumulation and role of IL-2 receptor-binding

SUPPLEMENTARY MATERIALS

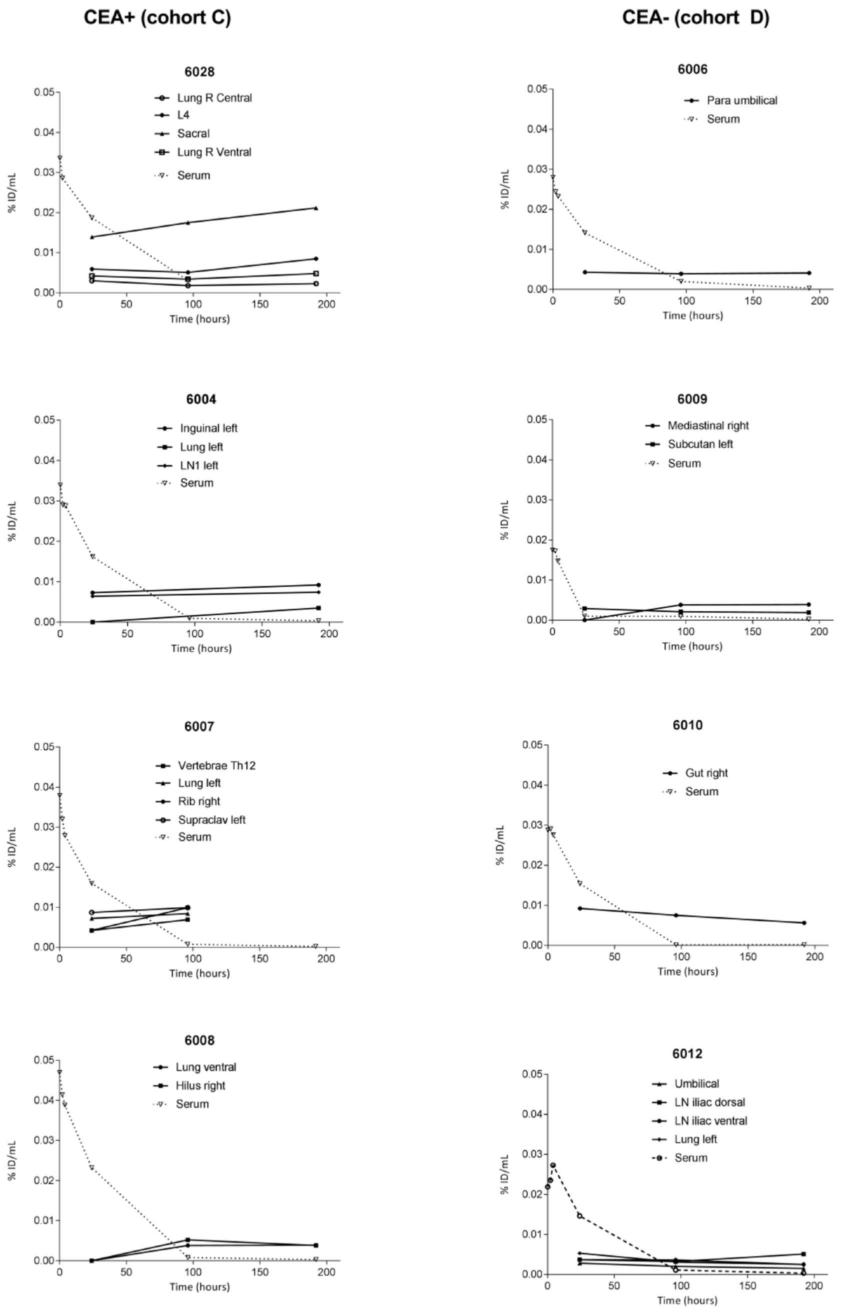

Supplementary Figure 1: ^{89}Zr-CEA− IL2v tumor uptake and serum levels of over time in CEA+ and CEA− patients treated with 30 mg. Data for individual patients from cohort C (CEA+; *n* = 4) and cohort D (CEA−; *n* = 4); tumor accumulation (solid lines) is $\%ID/mL_{peak}$ and serum pools (dashed line) is %ID/mL.

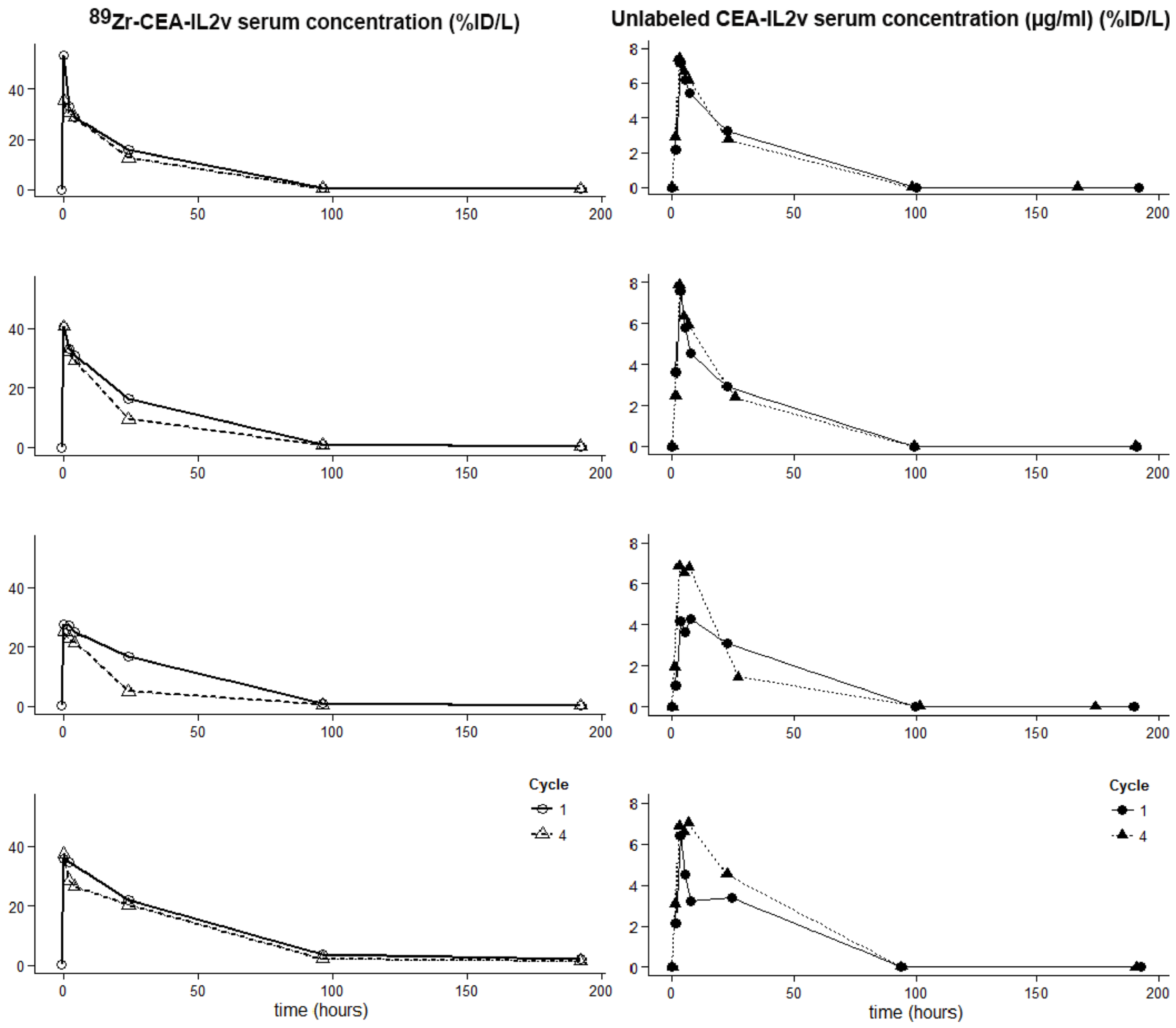

Supplementary Figure 2: ^{89}Zr-CEA− IL2v and unlabeled CEA− IL2v exposure over time. Individual serum concentration–time curves of ^{89}Zr –CEA− IL2v in % injected dose/mL (%ID/mL; open symbols) and of unlabeled CEA− IL2v (µg/mL) (filled symbols) in cycle 1 (solid line) vs. cycle 4 (dashed line) for four patients treated with 20 mg in the first q2w cycle and 30 mg thereafter (cohort E, CEA+ tumors). The patient represented in the third row had detectable anti-drug antibodies at cycle 4.

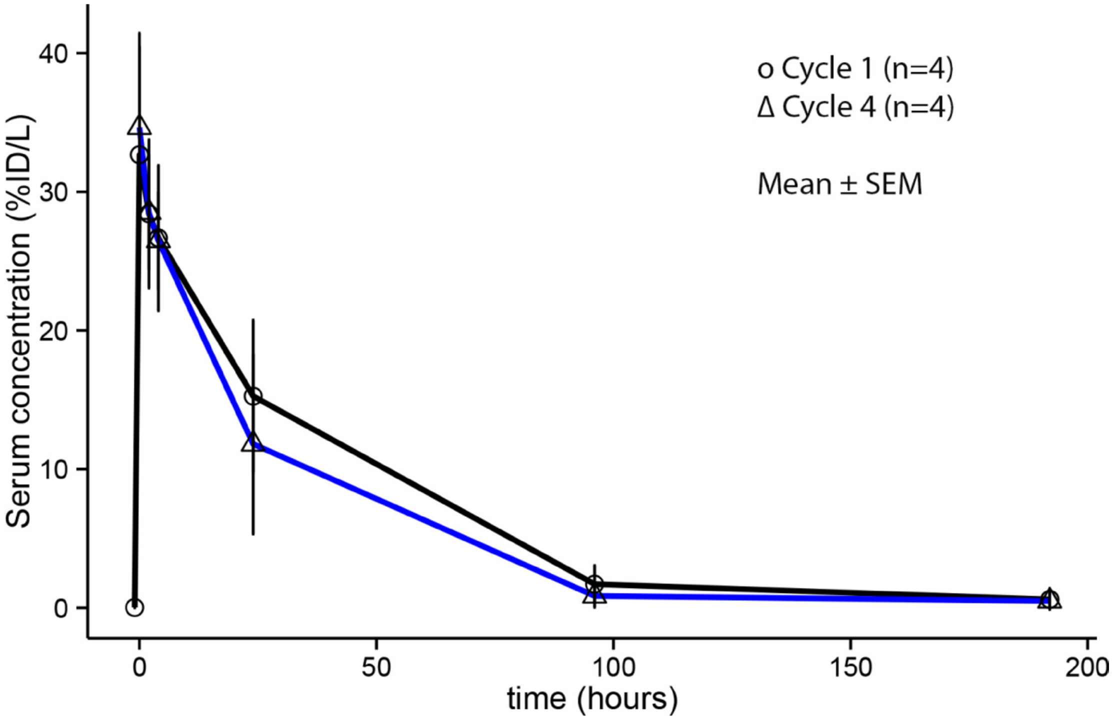

Supplementary Figure 3: ^{89}Zr-CEA− IL2v exposure at cycle 1 and cycle 4 in CEA+ patients from cohort E (*n* = 4). Multiple drug administration induced faster clearance of CEA− IL2v, reducing drug exposure.

Supplementary Table 1: Patient characteristics

Demographic characteristic	Value (*N* = 17)
**Age (years) [range]**	65 [42–78]
**Gender (%)**	
**Male**	58%
**Female**	42%
**ECOG PS (%)**	
**0**	43%
**1**	57%
**Tumor type (*n*) *(%)***	
CEA+	17
**CRC**	11 (46%)
**NSCLC**	4 (17%)
**Salivary gland**	1 (4.2%)
**Gastric**	1 (4.2%)
CEA−	7
**RCC**	3 (13%)
**Melanoma**	2 (8.3%)
**Ovarian**	1 (4.2%)
**Pancreatic cancer**	1 (4.2%)

ECOG PS = Eastern Cooperative Oncology Group Performance Status; CRC = colorectal carcinoma; NSCLC = non-small cell lung carcinoma; RCC = renal cell carcinoma.

Supplementary Table 2: Tumor accumulation; assessment of ^{89}Zr-accumulation in ^{18}F-FDG+ lesions per patient

Patient/ Cohort		Dose cycle 1 (mg)	CEA status	No. of FDG +ve lesions	No. of ^{89}Zr +ve lesions day 5	Accumulation visually confirmed	Consistency
1/A		6	CEA+	7	1	Yes	Negative on day 2
2/A		6	CEA+	5	1	Yes	Positive on day 2
3/A		6	CEA+	5	0	No	Consistent lack of accumulation
4/A		6	CEA+	11	7	Yes	Consistent
5/B		6	CEA−	9	2	No	Both negative on day 1 and 2
6/B		6	CEA−	6	0	No	Consistent lack of accumulation
7/B		6	CEA−	7	0	No	Consistent lack of accumulation
8/C		30	CEA+	7	5[a]	Yes	Positive on day 2 and 9, day 5 scan not done
9/C		30	CEA+	4	4	Yes	Consistent
10/C		20	CEA+	8	2	Yes	Consistent
11/ C		30	CEA+	10	6	Yes	Consistent
12/C		30	CEA+	8	4	Yes	Positive on day 5 and 9.
13/ D		30	CEA−	7	2	Yes	One lesion negative on day 2 (lymph node)
14/D		30	CEA−	2	1	Yes	Consistent
15/D		30	CEA−	8	1	Yes	Consistent
16/D		30	CEA−	6	4	Yes	Consistent
17/E		20	CEA+	4	0	No	Consistent lack of accumulation
18/E		20	CEA+	33	27	Yes	Consistent
19/E		20	CEA+	7	7	Yes	Consistent
20/E		20	CEA+	2	Not evaluable	Not evaluable	Note: Lack of FDG+ extrahepatic lesions
21/E	Cycle 1	20	CEA+	7	6	Yes	Consistent
	Cycle 4			7	4	Yes	Consistent
22/E	Cycle 1	20	CEA+	3	1	Yes	Consistent
	Cycle 4			3	1	Yes	Consistent
23/E	Cycle 1	20	CEA+	8	7	Yes	Consistent
	Cycle 4			8	3	Yes	Consistent
24/E	Cycle 1	20	CEA+	4	1	Yes	Consistent
	Cycle 4			4	1	Yes	Consistent

Accumulation was visually confirmed if ^{89}Zr-positive lesions were visual at the cycle 1, day 5 post-infusion plus one additional scan. [a]Primary evaluation at cycle 1, day 2 post-infusion because day 5 post-infusion scan was not performed.

Supplementary Table 3: Biodistribution per dose cohort and by CEA status on cycle 1, day 5 post-infusion (non-tumor tissue)

Organ (%ID/mLmean ±SD * 10^{-2})	6 mg		20 mg[a]	30 mg		Total		Overall
	CEA+ (*n* = 4)	CEA− (*n* = 3)	CEA+ (*n* = 9)	CEA+ (*n* = 4)	CEA− (*n* = 4)	CEA+ (*n* = 17)	CEA− (*n* = 7)	
**Spleen**	1.4 ± 0.23	1.6 ± 0.51	1.2 ± 0.30	0.78[b] ± 0.54	0.94[b] ± 0.25	1.2 ± 0.40	1.2 ± 0.49	1.2 ± 0.42
**Liver**	1.1 ± 0.32	0.94 ± 0.22	1.2 ± 0.27	0.90 ± 0.65	1.1 ± 0.25	1.1 ± 0.39	1.01 ± 0.23	1.1 ± 0.35
**Vertebrae**	0.41 ± 0.11	0.46 ± 0.044	0.49 ± 0.15	0.40 ± 0.28	0.42 ± 0.086	0.45 ± 0.17	0.44 ± 0.068	0.44 ± 0.15
**Kidneys**	0.36 ± 0.058	0.39 ± 0.055	0.36 ± 0.070	0.26 ± 0.18	0.30 ± 0.015	0.34 ± 0.10	0.34 ± 0.059	0.34 ± 0.092
**Lung**	0.10 ± 0.010	0.10 ± 0.032	0.10 ± 0.030	0.072 ± 0.049	0.11 ± 0.024	0.10 ± 0.033	0.11 ± 0.026	0.10 ± 0.031

[a]Including the 20 mg (*n* = 1) and 20–30 mg cohort (*n* = 8).

[b]Significant difference ($p < 0.05$) assessed by One Way Analysis of Variance (ANOVA) comparing $\%ID/mL_{mean}$ across 6 mg vs. 20 mg vs. 30 mg per organ and for CEA + vs. CEA− per organ.
